# Study on The Anti-Inflammatory Effects of *Callicarpa nudiflora* Based on The Spectrum–Effect Relationship

**DOI:** 10.3389/fphar.2021.806808

**Published:** 2022-01-27

**Authors:** Yamei Li, Yifang Yang, Xingdong Kang, Xiaofeng Li, Yongzhong Wu, Junping Xiao, Yang Ye, Jianqiong Yang, Yang Yang, Hai Liu

**Affiliations:** ^1^ The Clinical Medicine Research Center of the First Clinical Medical College, Gannan Medical University, Ganzhou, China; ^2^ College of Pharmacy, Gannan Medical University, Ganzhou, China; ^3^ China State Institute of Pharmaceutical Industry Shanghai Institute of Pharmaceutical Industry, Shanghai, China; ^4^ Shanghai Yaochen Biotechnology Co. Ltd., Shanghai, China; ^5^ School of Chinese Materia Medica, Nanjing University of Chinese Medicine, Shanghai, China; ^6^ Jiangxi Puzheng Pharmaceutical Co. Ltd., Ji’an, China

**Keywords:** *Callicarpa nudiflora*, fingerprint, anti-inflammatory, spectrum–effect relationship, natural compounds

## Abstract

*Callicarpa nudiflora* (*C. nudiflora*) is widely used to treat inflammation-related diseases in China. *C. nudiflora* mainly contains phenylethanol glycosides, flavonoids, triterpenes, diterpenes, iridoid glycosides, volatile oils, and other small molecules. Therefore, it is necessary to screen out anti-inflammatory active substances from *C. nudiflora*. In this paper, high-performance liquid chromatography was used to establish the fingerprint of *C. nudiflora* extracts. The anti-inflammation of *C. nudiflora* extracts were evaluated by the experiment of toes swelling in inflammatory rats. Then, the spectrum–effect relationship between the fingerprints and anti-inflammatory activities was researched by Pearson analysis and orthogonal partial least squares analysis to identify a group of anti-inflammatory compounds of *C. nudiflora* extracts. The differences of extracts are illustrated by principal component analysis and cluster analysis in pharmacological effects. Finally, 12 compounds, including catalpol (P1), caffeic acid (P2), protocatechuic acid (P9), 3,4-dihydroxybenzaldehyde (P10), forsythiaside E (P12), protocatechualdehyde isomers (P14), forsythiaside B (P15), rutin (P16), alyssonoside (P21), verbascoside (P22), 2′-acetyl forsythoside B (P24), and isorhamnetin (P32) by HPLC-DAD and UPLC-Q-TOF MS/MS, were determined as potential compounds for anti-inflammatory activity in *C. nudiflora*. In particular, six compounds were identified as active substances with the greatest anti-inflammatory potential. Moreover, all compounds were tested for anti-inflammatory experiments or anti-inflammatory literature retrieval. In this study, a method for rapid screening of potential anti-inflammatory active ingredients of *C. nudiflora* was established, which can provide a reference for the future study of active compounds of *C. nudiflora*.

## Introduction

The dry leaves or leafy shoots of *Callicarpa nudiflora* Hook. et Arn., belonging to the family Verbenaceae, are used as medicine for treating inflammation-related diseases. There are about 190 species of *Callicarpa nudiflora* worldwide, of which 46 species grow in China. *C. nudiflora* was commonly used as traditional ethnic medicine in Hainan because of growing in Li nationality area, and was also called Li medicine (Cai et al., 2012). Its chemical composition has been reported to be mainly flavonoids, phenylethanol glycosides, terpenes, volatile oils, etc. (Yi et al., 2019; Yang et al., 2021), which had anti-inflammatory, detoxification, astringency, and hemostasis functions. It has been widely applied to treat purulent inflammation, acute infectious hepatitis, respiratory and digestive tract bleeding, and traumatic bleeding (Wang et al., 2008; Tu et al., 2013; Wang, 2014).

*C. nudiflora* has great anti-inflammatory potential. According to the reports, Wang et al. found that four new 3,4-seco-labdane diterpenoids and other four known compounds from *C. nudiflora* showed significant inhibitory effects against NO production compared to the positive control dexamethasone (Wang HG. et al., 2019). The crude extract and the separated parts of *C. nudiflora* were confirmed to possess good anti-inflammatory effects (Zhang et al., 2013; Yang et al., 2015). Liang et al. found that the total flavonoids may be the material basis for the anti-inflammatory effects of *C. nudiflora* (Liang et al., 2009)*.* Moreover, the proprietary Chinese medicine—granules of *C. nudiflora*, sold on the market, was often used to treat inflammation-related diseases, such as acute tonsillitis, acute hepatitis, and bacterial pneumonia. Even so, due to the complexity of the chemical composition of *C. nudiflora*, the material basis of anti-inflammatory effect is difficult to clarify.

Chromatographic fingerprints reveal the chemical characteristics and evaluate the quality of traditional Chinese medicine (TCM) as a whole. The features of chromatographic fingerprints were characteristic, specificity, stability, and completeness. However, the fingerprint spectrum only elucidated the chemical composition, and internal connection between the chemical composition and efficacy was not clarified (Wei and Liu, 2019; Zhang et al., 2019; Zhu et al., 2019). Therefore, chemometrics were used to establish a relationship between fingerprints and efficacy, which is called spectrum–effect relationship research. Fingerprints provide information of the chemical components of TCM, while pharmacological research provides information of their activities. Then, the spectrum–effect correlation studies can illustrate the material basis of TCM more effectively (Zeng et al., 2015; Zhu et al., 2016). This method can quickly narrow the screening range of active substances in TCM, which has been confirmed by a large amount of literature. Liu et al. found three main active ingredients corresponding to the anti-inflammatory pharmacological effects of Farfarae Flos by using the spectrum–effect relationship (Yang L. et al., 2020). Four active compounds (caffeic acid, salvianolic acid B, hydroxysafflor yellow A, and lithospermate acid) have a good blood-activating effect, which is predicted by spectrum–effect relationship analysis (Wang YL. et al., 2019). In this paper, we will explore the possible material basis of the anti-inflammatory effect of *C. nudiflora* based on the spectrum–effect relationship.

In this study, firstly, the fingerprints of the six extracts of *C. nudiflora* were established. Secondly, the rat inflammation model was used to evaluate the anti-inflammatory effect. Next, Pearson correlation analysis, orthogonal partial least squares (OPLS) analysis, principal component analysis (PCA), and cluster analysis were used to study the correlation between the HPLC fingerprints of six extracts and their anti-inflammatory activities. Finally, we conducted inhibition experiments of COX and anti-inflammatory literature searches on the selected compounds.

## Materials and Methods

### Instruments

The following instruments were used: Type 1260 high-performance liquid chromatography (Agilent, Santa Clara, CA, United States), Waters Acquity UPLC/Xevo®G2-XS QTOF ultra-high performance liquid chromatography tandem quadruple time-of-flight mass spectrometer (Waters Corp., Milford, MA, United States), SPSS 22.0 statistical analysis software, FA1004 electronic balance (Shanghai Jingke Balance Factory, Shanghai, China), thickness gauge (Shanghai Liuling Instrument Factory, Shanghai, China), 96-hole blackboard (Corning Inc., Corning, NY, United States), imported gun head (Axygen, Union City, CA, United States), and Biotek Synergy H1 microplate reader (Agilent).

### Materials and Reagents

*C. nudiflora*, harvested from July to September in Baisha and Wuzhishan of Hainan Province, China, was purchased from Jiangxi Puzheng Pharmaceutical Co., Ltd., Jiangxi, China, and identified as *Callicarpa nudiflora* Hook. et Am. by researcher Wu Yongzhong. Sodium chloride was purchased from Nanjing Chemical Reagent Co., Ltd., Jiangsu, China (batch number: 140709); dexamethasone acetate tablets, from Zhejiang Xianju Pharmaceutical Co., Ltd., Zhejiang, China (batch number: 150317, 0.75 mg/tablet); granules of *C. nudiflora*, from Jiangxi Puzheng Pharmaceutical Co., Ltd. (batch number: 150512, 3 g/bag); carrageenan, from Sigma-Aldrich, St. Louis, MO, United States (batch number CAS9000-07-1); Cox fluorescent inhibitor screening assay kit, from Cayman Chemical (Ann Arbor, MI, United States); and verbascoside (batch number M-001-170315), forsythiaside B reference substance (batch number L-013-170421), and luteolin reference substance (batch number PRF8030242) from Chengdu Refines Biotechnology Co., Ltd., Chengdu, China. All quality control scores were greater than 98%.

Acetonitrile and phosphoric acid (chromatographically pure) were obtained from ACS, Washington, DC, United States; ethanol and methanol (analytically pure), from Shanghai Titan Technology Co., Ltd., (Shanghai, China). Water was self-made double-distilled water.

### Animals

SD rats (180–220 g), male and female half, were purchased from the Experimental Animal Center of Nantong University (Hunan, China). All rats were housed under standard conditions of temperature and humidity with a 12-h light and dark cycle and fed a standard pellet diet and water ad libitum. The animal study was performed according to the international rules considering animal experiments and the internationally accepted ethical principles for laboratory animal use and care. Animal welfare and experimental procedures were carried out following the ethical regulations of Gannan medical university [certificate number: SYXK (Gan) 2014-0001].

### HPLC-DAD and UPLC-Q-TOF MS Conditions

HPLC-DAD analysis was performed on the Agilent LPLC-1260 system. Chromatographic separation was conducted on a Welch Material C_18_ column (4.6 mm × 250 mm, 5 μm); the mobile phase consists of 0.5% phosphoric acid (A) and acetonitrile (B) with a gradient elution as –follows: 0–10 min, 82% A; 10–20 min, 80% A; 20–30 min, 76% A. The flow rate was maintained at 1 ml/min and the column temperature was set at 35°C. The detector belongs to UV-DAD. The detection wavelength was set at 330 nm. The injection volume was set at 10 μl.

The UPLC-Q-TOF-MS analysis was performed on the Waters Acquity UPLC/Xevo®G2-XS QTOF ultra-high performance liquid chromatography tandem quadruple time-of-flight mass spectrometer (Waters). The chromatographic column was 1.8 μm Ultimate XB-C_18_ column (100 mm × 2.1 mm i.d.). The mobile phases were water (A) and acetonitrile (B) with a gradient as follows (Li et al., 2020): 0–8 min, 98% A; 8–13 min, 80%; 13–23 min, 55%; 23–36 min, 2% A; 36–40 min, 98% A. The flow rate was maintained at 0.25 ml/min and column temperature was set at 30°C. The MS spectrometry conditions were as follows: ESI ion source, negative ion detection mode; detection conditions were as follows: capillary voltage, 2 kV; cone voltage, 30 V; extraction cone voltage, 6 V; ion source temperature, 120°C; dissolvent gas temperature, 500°C; cone gas flow velocity, 50 L/h; dissolvent airflow velocity, 900 L/h; collision energy, 6.0 V. Data were collected and analyzed with UNIFI software (v 18.2.0, Waters Corp.). Furthermore, the fragment information of the obtained secondary mass spectrometry was compared with related literature and mass spectrometry database websites for in-depth identification. Related mass spectrometry databases are as follows: Chemspider (http://www.chemspider.com/), HMDB (http://www.hmdb.ca/), METLIN (http://metlin.scripps.edu/), and chemical components of Traditional Chinese medicine database (http://unpd.chem960.com/).

### Preparation of Sample Solutions

The dry leaves of *C. nudiflora* were crushed into powders and sieved through No. 4 sieves.

Sample solution 1: Three copies *C. nudiflora* medicinal materials were extracted with 10 times amount of water, 60% ethanol, and 95% ethanol by the heat reflux extraction within 1 h, repeated two times. The combined filtrate was recovered under reduced pressure to dryness to obtain extract 1 (1 g extract powder was equivalent to 3.62 g of the raw herbs), extract 2 (1 g extract powder was equivalent to 2.82 g of the raw herbs), and extract 3 (1 g extract powder was equivalent to 3.85 g of the raw herbs). Part of the extract two was dissolved in an appropriate amount of water, passed through an AB-8 macroporous adsorption resin column, first eluted with water until the eluent was clear, then changed to 40% ethanol to continue elution until the eluent was clear, and then continued with 60% ethanol to after elution until the eluate was clear. The combined eluates were collected separately and recovered to dryness under reduced pressure to obtain extract 4 (1 g extract powder was equivalent to 7.17 g of the raw herbs), extract 5 (1 g extract powder was equivalent to 7.07 g of the raw herbs), and extract 6 (1 g extract powder was equivalent to 55.56 g of the raw herbs). The extract powder of the six extracts (equivalent to 1 g of the raw herbs) was accurately weighed in an eggplant-shaped bottle and added with 50 ml 50% methanol by shaking well. Then, they were respectively filtered and collected.

Sample solution 2: The medicinal powder of *C. nudiflora* was accurately weighed for 1 g in eggplant-shaped bottle and added with 50 ml 50% methanol, then extracted by refluxing for 1 h and cooled; 50% methanol was used to make up for the lost weight after re-weighing. The sample was obtained by filtering and collecting the filter fluid.

### Establishment of Fingerprint

Software Similarity Evaluation System for Chromatographic Fingerprint of TCM (2012 version) was used to obtain fingerprints and verify their precision, repeatability, and stability. At the same time, it was also used to provide the relative retention time and relative peak area of the chromatographic peak. The fingerprint pattern generated by Sample Solution 2 was used as a reference fingerprint pattern. The fingerprints of the six extracts were derived from Sample Solution 1. Then, all the fingerprints were automatically matched by the median multipoint correction method. Precision testing: six consecutive sample injections using the same method; repeatability testing: samples are repeatedly measured six times; stability: samples are measured at 0, 2, 4, 6, 12, and 24 h.

### Experiments of Pharmacodynamic Effects

In this paper, six extracts of *C. nudiflora* were used to conduct anti-inflammatory experiments on inflammation model rats.

#### Preparation of Gavage Reagent

The concentration of dexamethasone acetate tablets was prepared at 0.1 mg/ml (prepared for current using). The concentration of granules of *C. nudiflora* was prepared at 0.4 g/ml. The concentrations of six extracts all were prepared to 0.2 g/ml. All the above reagents were 20 times the clinical human dose (4 g/kg) and administered by intragastric administration of 1 ml/100 g.

#### Grouping and Methods of Experiment

All rats were normally fed for 7 days. After that, they were divided into control group (saline), positive control drug group 1 (dexamethasone acetate tablet), positive control drug group 2 (granules of *C. nudiflora*), and groups of six extracts with 10 rats in each group (*n* = 10). All drugs were administered through gavage once daily for 7 consecutive days; 0.1 ml of 1% carrageenan suspension was subcutaneously injected into the left hind limb of the rats in 0.5 h after the last administration. After inflammation, the thickness of the toe was measured with a thickness gauge every 1 h for six consecutive times, calculating the swelling inhibition rate.

### The Spectrum–Effect Relationship Study

#### Pearson Correlation Analysis

Pearson correlation analysis is a method of parameter, which is widely used to measure the linear relationship between variables. In this study, the chromatographic data and anti-inflammatory efficacy data of six extracts were imported into the SPSS 22.0 analysis software, and then their correlations were analyzed by this method. In addition, the correlation of scatter plot based on the data obtained from the peak area of the chromatogram peak and the efficacy was made for the significantly correlated chromatographic peaks that have been screened out.

#### Orthogonal Partial Least Squares Analysis

OPLS is a variant of partial least squares (PLS) which uses orthogonal signal correction to maximize the explained covariance between *X* and *Y* on the first latent variable, and components >1 capture variance in *X* which is orthogonal (or unrelated) to *Y*. It is emphasized that OPLS does not change predictive power compared with PLS, given that the model complexity is the same. It is often used when attempting to understand the relationship between the raw data and the process. In this case, chromatographic peak variables (*X*) and efficacy data variables (*Y*) were imported into SIMCA 14.1 (Umetrics, Sweden) for OPLS analysis.

#### Principal Component Analysis

PCA is a widely used data dimensionality reduction algorithm. It uses orthogonal transformation to convert multiple variables into a set of new variables, thereby displaying the characteristics of the data in smaller latitude. When the research has multiple indicators or variables, it is usually used because there are some correlations and data overlaps between them and it is difficult to study the distribution of samples in high-dimensional space. PCA adopts a dimension reduction method to find a set of comprehensive factors to represent the original variables, thus simplifying the analysis. In this paper, the 22 correlation peaks of the six extracts were imported into SPSS 22.0 for PCA.

#### Cluster Analysis

Cluster analysis is classifying similar research objects while maximizing the sameness between similar objects and the difference between heterogeneous objects. In this paper, the relevant data of the six extracts were imported into SPSS 22.0 for cluster analysis.

### Effect on Activity of Cyclooxygenase (COX-1 and COX-2)

According to the instructions on the kit, the six extracts, the granules of *C. nudiflora*, and the active compounds were detected in cyclooxygenase (COX-1 and COX-2) activity and detected at Ex: 530–540 nm and Em: 585–590 nm, respectively. The samples to be tested were formulated into different concentration gradients. The inhibition rate and IC_50_ of different samples for COX-1 and COX-2 were obtained by detecting the changes of fluorescence intensity after adding the samples.

## Results

### HPLC Fingerprints

**FIGURE 1:**
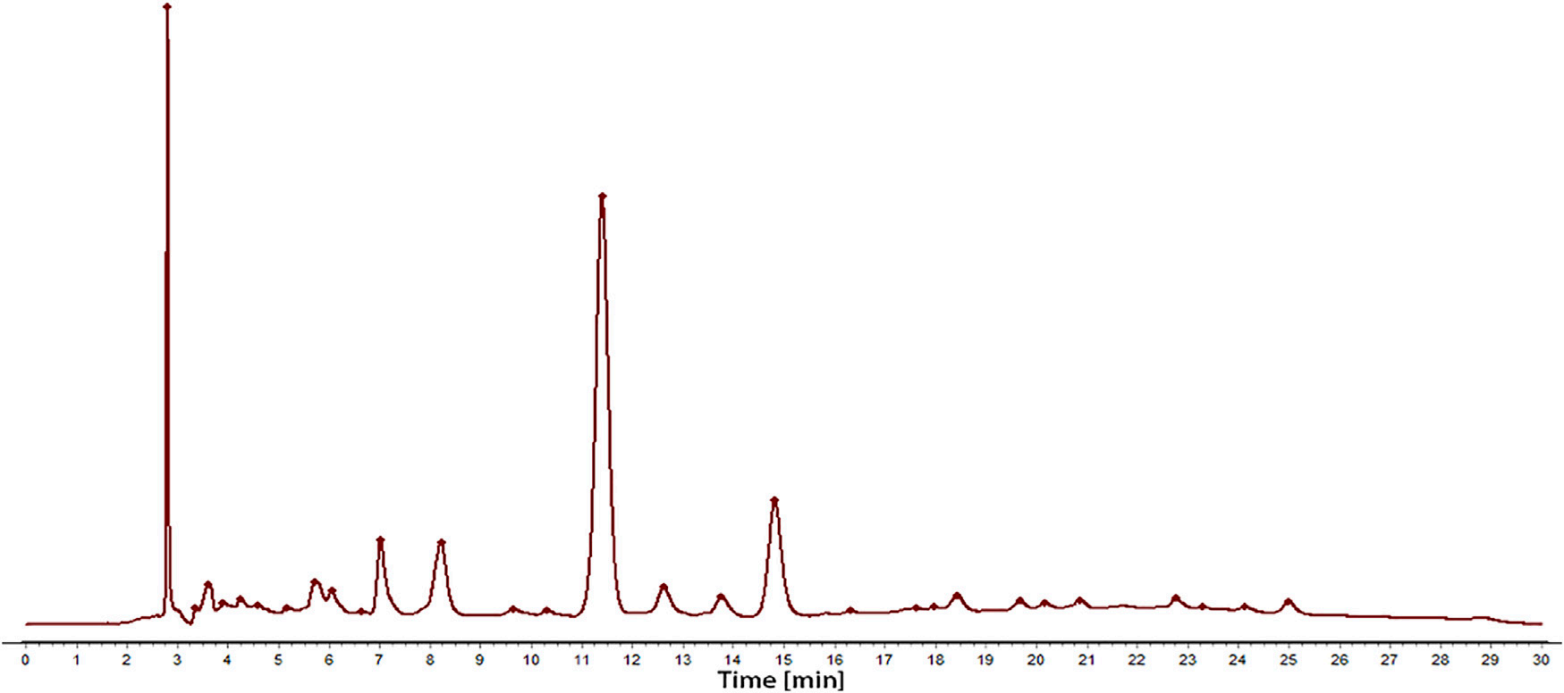
The chromatogram of the total extract obtained by conventional decocting (water) of *C. nudiflora* leaves.

**FIGURE 2:**
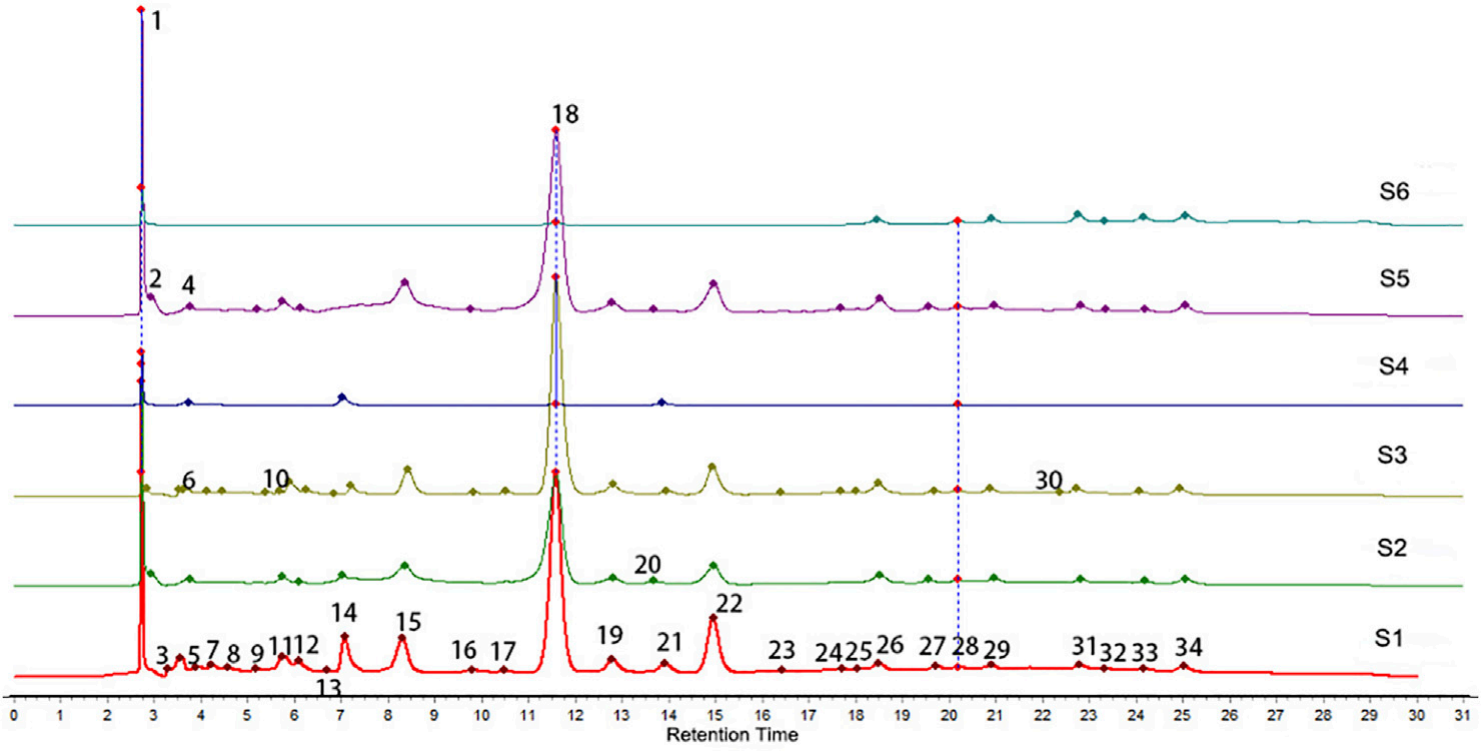
HPLC chromatogram overlay and feature peaks of six extracts.

Sample solution 2 was regarded as a control fingerprint though the methods of median multipoint correction and the chromatographic peaks automatically matched, as shown in Figure 1. Sample solution 1 was compared with the control fingerprints, processed in the same way. Thirty-four fingerprint peaks were screened out, as shown in Figure 2. Then, 22 peaks with a matching number greater than 2 were extracted from these for spectral efficiency correlation analysis (as shown in Supplementary Table S1).

In addition, the results of the methodological study demonstrated that RSD values of the relative retention of each common peak were less than 0.2%, and RSD values of the relative peak area were less than 1.8%, which indicates that the precision of the method is good. Besides, inspecting their repeatability and stability found that RSD values of the relative retention time of each common peak were less than 0.2%, and RSD values of the relative peak area were less than 2.3% and 2.7% respectively, which indicated that it has good repeatability of the method and stability of the sample solution; after analyzing the similarity of HPLC fingerprints of all batches of *C. nudiflora*, the similarities were higher than 0.9, which demonstrated that the quality of *C. nudiflora* was stable. So, the fingerprint of the herbs can be used as a control for the HPLC spectra of six extracts.

### Anti-Inflammatory Activity

The thickness of the inflamed rats’ toes was measured at different time points as the anti-inflammatory indicators of all samples. We found that all six extracts showed inhibitory effects on the swelling of the feet of rats. However, extracts 1, 2, 3, and 5 had greater inhibition effects than the control group significantly, the effects of extracts 4 and 6 are relatively small, as shown in Table 1. At the same time, by calculating the inhibition rate of all samples to toe swelling of rats, as shown in Table 2, the difference in the efficacy of the six extracts may be caused by the difference in chemical composition between them. Therefore, it is necessary to study the relationship between the six extracts of *C. nudiflora* and the anti-inflammatory activity and to find the potential anti-inflammatory components through the spectrum–effect relationship analysis.

**TABLE 1:** Effect of different *C. nudiflora* extracts on carrageenan-induced swelling of rats ( $\bar{X}\pm s$ ).

Group	Swelling degree (mm) at different times after inflammation (h)					
	1 h	2 h	3 h	4 h	5 h	6 h
Blank group	2.91 ± 1.07	3.54 ± 0.86	4.03 ± 0.92	4.24 ± 0.87	4.27 ± 0.82	4.12 ± 0.49
Dexamethasone acetate group	1.27 ± 0.71**	1.63 ± 1.33**	1.78 ± 0.84**	2.52 ± 0.91**	2.56 ± 1.05**	2.19 ± 0.88**
Granules of C. nudiflora	1.55 ± 0.79**	2.75 ± 0.91	2.87 ± 0.54**	3.64 ± 0.86	3.76 ± 0.56	3.54 ± 0.72
Extract 1 group	1.83 ± 0.70*	2.25 ± 0.57**	2.66 ± 0.61**	2.74 ± 1.00**	3.64 ± 0.68	3.52 ± 0.65**
Extract 2 group	1.59 ± 0.71**	2.87 ± 0.51*	3.49 ± 1.03	3.62 ± 0.64	3.84 ± 0.45	3.73 ± 1.15
Extract 3 group	1.99 ± 0.51*	2.55 ± 1.15*	3.80 ± 1.00	3.55 ± 1.02	3.79 ± 0.89	3.75 ± 0.96
Extract 4 group	2.65 ± 1.02	3.57 ± 0.78	3.72 ± 1.31	3.76 ± 1.04	3.82 ± 0.88	3.83 ± 0.78
Extract 5 group	1.57 ± 0.67**	2.66 ± 0.68*	3.55 ± 1.00	3.44 ± 1.12	3.77 ± 0.73	3.88 ± 0.91
Extract 6 group	2.25 ± 0.45	2.79 ± 0.46*	3.80 ± 0.75	3.90 ± 0.55	3.95 ± 0.82	3.83 ± 0.95

Compared with the blank group *p < 0.05, **p < 0.01.

**TABLE 2:** Effect of different *C. nudiflora* extracts on inhibition rate of carrageen-induced swelling of the foot in rats.

Group	Inhibition rate (%) at different times after inflammation (h)					
	1 h	2 h	3 h	4 h	5 h	6 h
Dexamethasone acetate Group	56.4	53.9	56.0	40.5	40.1	46.7
Granules of *C. nudiflora*	46.6	22.3	28.9	14.1	11.8	14.0
Extract 1 group	37.1	36.4	34.0	35.3	14.8	14.5
Extract 2 group	45.2	18.9	13.5	14.7	10.1	9.3
Extract 3 group	31.6	28.0	5.7	16.4	11.3	8.9
Extract 4 group	8.8	−0.9	7.8	11.3	10.4	6.8
Extract 5 group	46.0	25.0	12.0	18.9	11.7	5.6
Extract 6 group	22.7	21.1	5.9	8.0	7.5	7.0

### Pearson Correlation Analysis

**FIGURE 3:**
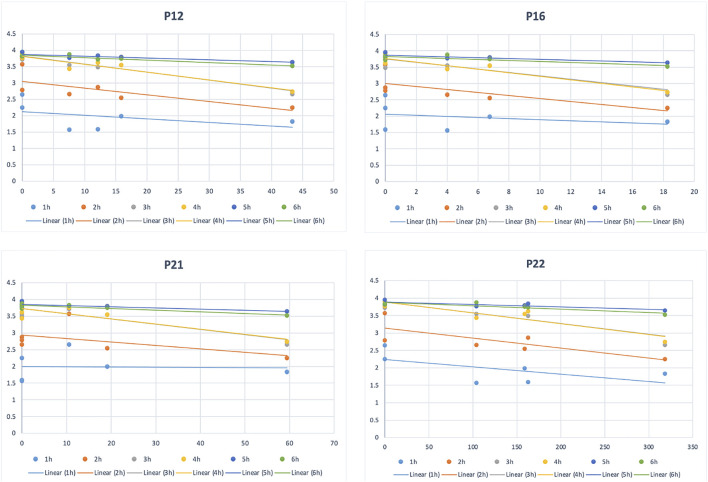
Scatterplot of some correlation peaks and drug efficacy data.

The results of Pearson correlation analysis show that the chromatographic peaks 1, 2, 9, 10, 12, 14, 15 (forsythiaside B), 16, 21, 22 (verbascoside), 24, and 32 were significantly associated with swelling of the toes of rats but negatively correlated, as shown in Table 3. Among them, chromatographic peaks 10, 12, 14, 15, 16, and 32 were particularly related to swelling (*p* < 0.01). At the same time, the negative correlation can also be seen in the correlation scatter plot, as shown in Figure 3.

**TABLE 3:** Pearson correlation analysis results.

Peak number	Pearson correlation analysis	Swelling of feet at different time points					
		1 h	2 h	3 h	4 h	5 h	6 h
P1	Correlation	−0.702	−0.465	−0.820*	−0.724	−0.624	−0.677
	Distinctiveness	0.120	0.352	0.046	0.104	0.185	0.140
P2	Correlation	−0.735	−0.027	0.072	0.105	0.081	0.176
	Distinctiveness	0.096	0.959	0.892	0.843	0.879	0.738
P5	Correlation	−0.515	0.220	0.104	0.201	0.148	0.154
	Distinctiveness	0.296	0.675	0.845	0.702	0.779	0.771
P9	Correlation	−0.357	−0.785	−0.742	−0.913*	−0.874*	−0.732
	Distinctiveness	0.487	0.064	0.091	0.011	0.023	0.098
P10	Correlation	−0.613	−0.610	−0.950**	−0.884*	−0.756	−0.814*
	Distinctiveness	0.195	0.199	0.004	0.019	0.082	0.049
P12	Correlation	−0.423	−0.745	−0.908*	−0.956**	−0.856*	−0.941**
	Distinctiveness	0.404	0.089	0.012	0.003	0.030	0.005
P14	Correlation	−0.252	−0.515	−0.924**	−0.873*	−0.772	−0.982**
	Distinctiveness	0.629	0.295	0.009	0.023	0.072	0.000
P15	Correlation	−0.911**	−0.686	−0.612	−0.721	−0.703	−0.447
	Distinctiveness	0.012	0.133	0.197	0.106	0.120	0.375
P16	Correlation	−0.291	−0.744	−0.855*	−0.954**	−0.875*	−0.844*
	Distinctiveness	0.576	0.090	0.030	0.003	0.022	0.035
P18	Correlation	−0.744	−0.703	−0.488	−0.640	−0.635	−0.615
	Distinctiveness	0.090	0.119	0.327	0.171	0.176	0.194
P19	Correlation	−0.699	−0.751	−0.679	−0.787	−0.742	−0.776
	Distinctiveness	0.123	0.085	0.138	0.063	0.092	0.070
P21	Correlation	−0.035	−0.540	−0.841*	−0.884*	−0.823*	−0.900*
	Distinctiveness	0.948	0.269	0.036	0.019	0.044	0.014
P22	Correlation	−0.612	−0.771	−0.844*	−0.913*	−0.833*	−0.882*
	Distinctiveness	0.196	0.073	0.034	0.011	0.040	0.020
P24	Correlation	−0.247	−0.751	−0.656	−0.835*	−0.801	−0.774
	Distinctiveness	0.636	0.086	0.157	0.039	0.055	0.071
P26	Correlation	−0.734	−0.657	−0.366	−0.524	−0.528	−0.534
	Distinctiveness	0.097	0.156	0.476	0.286	0.282	0.276
P27	Correlation	−0.767	−0.698	−0.764	−0.800	−0.727	−0.772
	Distinctiveness	0.075	0.123	0.077	0.056	0.102	0.072
P28	Correlation	−0.634	−0.672	−0.621	−0.755	−0.759	−0.744
	Distinctiveness	0.176	0.144	0.189	0.083	0.080	0.090
P29	Correlation	−0.776	−0.613	−0.457	−0.535	−0.500	−0.595
	Distinctiveness	0.069	0.195	0.363	0.274	0.313	0.213
P31	Correlation	−0.743	−0.708	−0.457	−0.590	−0.560	−0.619
	Distinctiveness	0.091	0.115	0.362	0.218	0.248	0.190
P32	Correlation	−0.396	−0.649	−0.899*	−0.918**	−0.809	−0.642
	Distinctiveness	0.437	0.163	0.015	0.010	0.051	0.169
P33	Correlation	−0.697	−0.697	−0.446	−0.559	−0.510	−0.651
	Distinctiveness	0.124	0.124	0.376	0.249	0.301	0.161
P34	Correlation	−0.803	−0.648	−0.478	−0.568	−0.532	−0.593
	Distinctiveness	0.054	0.164	0.338	0.240	0.277	0.215

*p < 0.05, **p < 0.01.

### Identification of 12 Related Peaks by UPLC-DAD-Q/TOF-MS

The compound identification was based on the relative retention time, UV maximum absorption, and the comparison of the mass fragment information through the UNIFI software. In addition, the chromatographic retention time and mass spectrometry data of some substances are compared with existing reference substances, including the compounds of *C. nudiflora* and *Callicarpa* L. that were separated and identified in our previous study (Yang J. Q. et al., 2020; Li et al., 2020). We determined each component’s relative molecular weight and then obtained fragmentation information based on the secondary mass spectrum. The 12 related peaks could be identified as catalpol, caffeic acid, protocatechuic acid, 3,4-dihydroxybenzaldehyde, forsythiaside E, protocatechualdehyde isomers, forsythiaside B, rutin, alyssonoside, verbascoside, 2′-acetyl forsythiaside B, and isorhamnetin, as shown in Table 4.

**TABLE 4:** Identification results of spectral efficiency related peaks by UPLC-DAD-Q/TOF-MS.

Peak number	Formula	Precise of molecular mass	MS^1^ adduct (*ms/z*)	MS^2^ (*ms/z*)	*λ*_max_	Compound
P1	C_15_H_22_O_10_	362.1213	[M + HCOO]^-^407.1189	197.8561 [M-H-Glc]^-^	384.5	Catalpol
			[M-H]^-^361.1133	150.9148 [M-H-Glc-CH_2_O-H_2_O]^-^		
P2	C_9_H_8_O_4_	180.0422	[M-H]^-^179.0341	179.0341 [M-H]^-^	218.0, 323.6	Caffeic acid
				135.0443 [M-H-CO_2_]^-^		
P9	C_7_H_6_O_4_	154.0266	[M-H]^-^153.0189	153.0189 [M-H]^-^	213.3, 318.8	Protocatechuic acid
				108.0211 [M-COO]^-^		
P10	C_7_H_6_O_3_	138.0317	[M-H]^-^137.0236	108.5072 [M-H-CHO]^-^	254.2, 324.8	3,4-dihydroxybenzaldehyde
				92.5135 [M-H-CHO-O]^-^		
P12	C_20_H_30_O_12_	462.1737	[M-H]^-^461.1688	461.1688 [M-H]^-^	281.9	forsythiaside E
				135.0435 [caffeoyl]^-^		
P14	C_7_H_6_O_3_	138.0317	[M-H]^-^137.0244	108.5046 [M-H-CHO]^-^	242.8, 327.2	protocatechualdehyde isomers
				92.5129 [M-H-CHO-O]^-^		
P15	C_34_H_44_O_19_	756.2476	[M-H]^-^755.2398	755.2398 [M-H]^-^	329.6	Forsythiaside B
				593.2086 [M-H- caffeoyl]^-^		
				179.0343; 161.0231; 135.0446 [caffeoyl]^-^		
P16	C_27_H_30_O_16_	610.1533	[M-H]^-^609.1456	609.1456 [M-H]^-^	254.2, 324.8	Rutin
				300.0271 [M-H-glu-*o*-rha]^-^		
P21	C_35_H_46_O_19_	770.2633	[M-H]^-^769.2548	769.2548 [M-H]-	254.6, 347.5	Alyssonoside
				593.2085 [M-H-caffeoyl]-		
P22	C_29_H_36_O_15_	624.205	[M-H]^-^623.1978	623.1978 [M-H]-	329.6	Verbascoside
				461.1659 [M-H- caffeoyl]-		
				161.0240 [caffeoyl]-		
P24	C_36_H_46_O_20_	798.2582	[M-H]^-^797.2506	797.2506 [M-H]^-^	328.4	2′-Acetyl forsythiaside B
				161.0239; 179.0346 [caffeoyl]^-^		
				133.02 [C_5_H_9_O_4_]^-^		
P32	C_16_H_12_O_7_	316.0583	[M-H]^-^315.0587	315.0587 [M-H]^-^	254.6, 349.9	Isorhamnetin
				151.0030 [C7H3O4]^-^		

### Orthogonal Partial Least Squares Analysis

**FIGURE 4:**
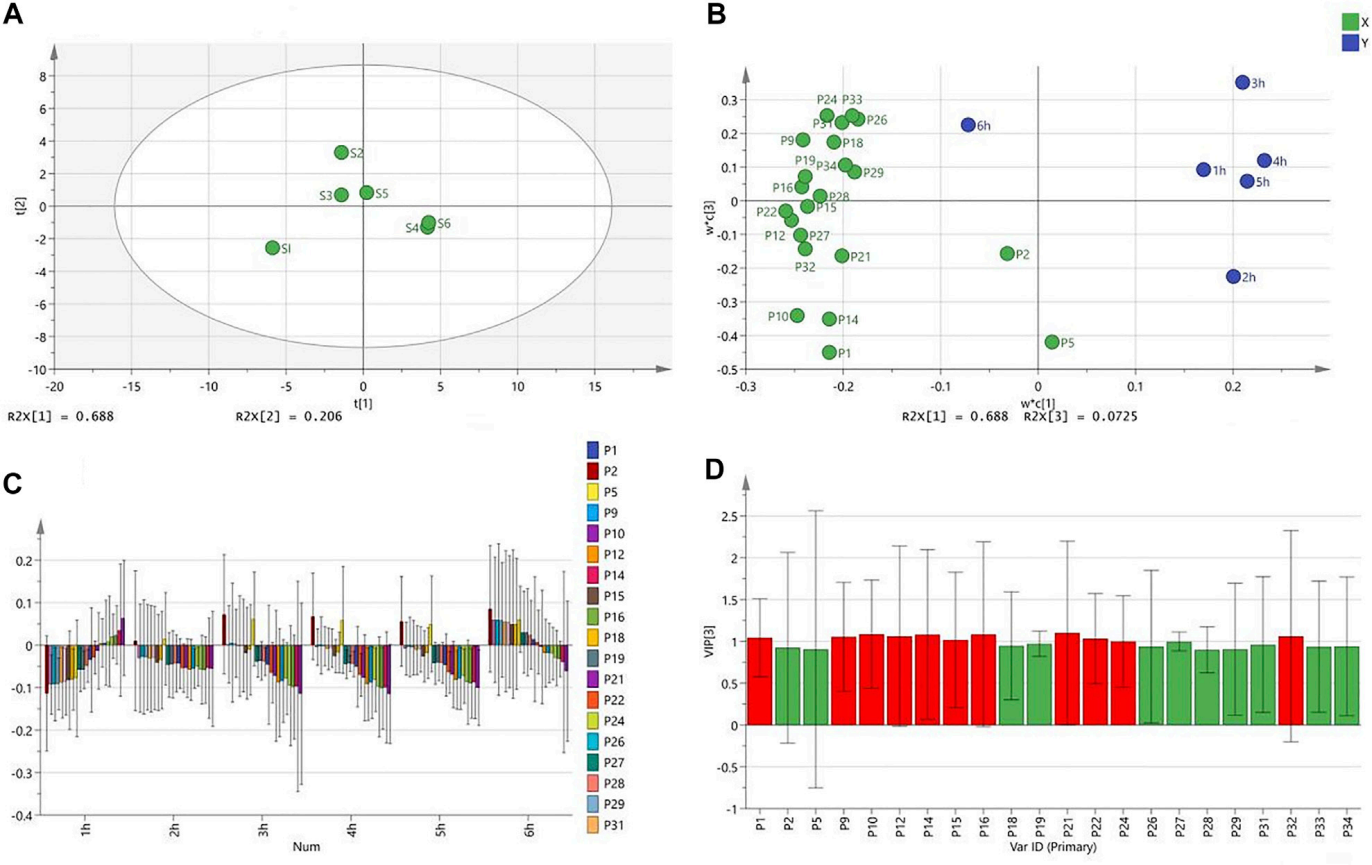
OPLS analysis results [**(A)** Score scatter Plot; **(B)** Loading Scatter Plot; **(C)** Coefficient overview Plot; **(D)** VIP value graph].

OPLS is a regression modeling method of multiple dependent variables to multiple independent variables. It can remove the data variation in the independent variable X that is not related to the categorical variable Y, so that the categorical information is mainly concentrated on one principal component. Therefore, simplifying and visualizing the model the effect is more obvious. The OPLS results are shown in Figure 4.

From the OPLS results, extract 1 was distributed in a single area, which had the greatest effect on the toe swelling of rats. Extracts 2 and 3, which had a greater effect, were distributed in the same area. Although extracts 3 and 5 are distributed in different regions, they are very close together. The worst-performing extracts, 4 and 6, were distributed in the same area and mostly overlap (Figure 4A). Furthermore, all chromatographic peaks were negatively correlated with the toes swelling of rats at different points in time except for 2 and 6 h (Figure 4B). Individual peaks are positively correlated with swelling (Figure 4C). The peaks marked red (VIP >1) were significantly related to the toe swelling of rats, and they were peaks 1, 2, 9, 10, 12, 14, 15, 16, 21, 22, 24, and 32 (Figure 4D), which are consistent with the results of Pearson correlation analysis.

### Principal Components Analysis

By means of extraction of the main ingredient, the contribution rate of the three main ingredients can be determined, which are 69.02%, 20.59%, and 7.22%, and the cumulative contribution rate of the three ingredients is 96.85%. This means that the cumulative total of these three main components explains 96.85% of the total variation.

In addition, in the component matrix, as shown in Table 5, ingredient 1 should be flavonoids and phenethyl alcohol glycosides based on luteolin and verbascoside, and ingredient 2 should be phenolic acid small molecule compounds with peak 2 (caffeic acid) and peak 5 as the main component; ingredient 3 should be iridoids with peak 1 (catalpol) as the main component. In conjunction with the results of Figure 4A, it found that many flavonoids, phenethyl alcohol glycosides, and phenolic acids were present in extracts 1, 2, 3, and 5. Differences in efficacy are caused by differences in the content or proportion of these three components. Many iridoids and other compounds were present in extracts 4 and 6, resulting in poor efficacy.

**TABLE 5:** Composition matrix.

	Ingredient		
	1	2	3
P1	0.793	0.201	0.567
P2	0.241	0.926	0.245
P5	0.133	0.925	0.303
P9	0.803	−0.525	−0.137
P10	0.852	−0.095	0.509
P12	0.919	−0.372	0.082
P14	0.821	−0.347	0.241
Forsythiaside B	0.85	0.259	0.188
P16	0.805	−0.588	0.013
P18	0.928	0.267	−0.252
Luteolin	0.989	0.095	−0.109
P21	0.703	−0.673	0.028
Verbascoside	0.99	−0.123	0.049
P24	0.79	−0.519	−0.319
P26	0.869	0.363	−0.335
P27	0.975	0.167	0.144
P28	0.967	0.107	−0.187
P29	0.884	0.438	−0.134
P31	0.911	0.305	−0.271
P32	0.647	−0.535	0.442
P33	0.897	0.298	−0.294
P34	0.9	0.414	−0.125

### Cluster Analysis

**FIGURE 5:**
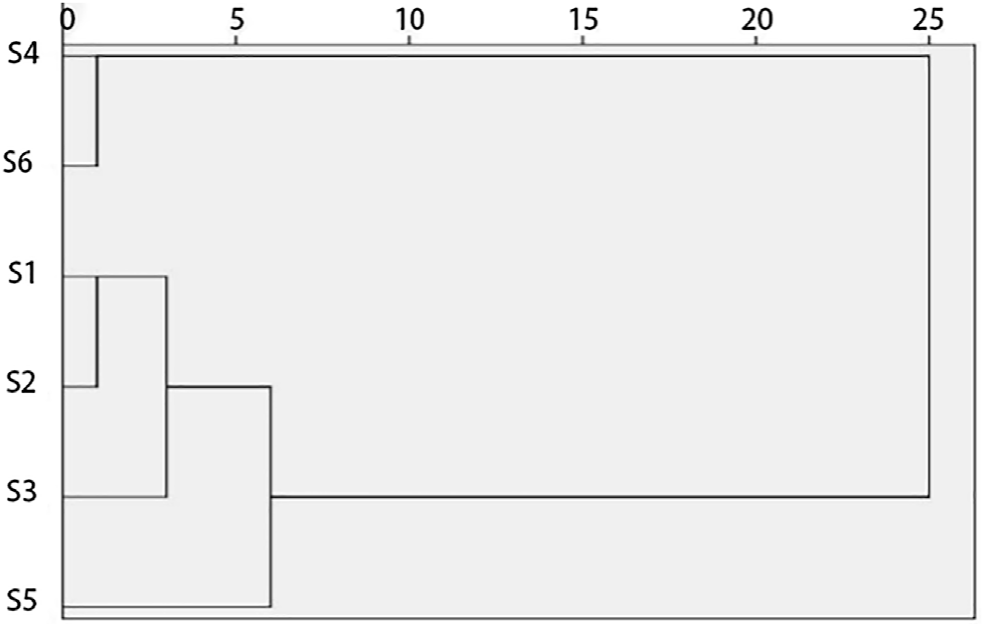
Cluster diagram of six extracts.

The inter-group link clustering analysis method in SPSS 22.0 software was used to verify the PCA, and its result is shown in Figure 5. The more effective extracts, 1, 3, 2, and 5, are gathered in one group, and the less effective extracts, 4 and 6, are gathered in another group. These results are consistent with the PCA results, which can explain that the result of PCA is reliable.

### Inhibitory Effect on Cyclooxygenase (COX-1 and COX-2)

The six extracts and their active compounds verbascoside, 2′-acetylforsythoside B, forsythoside B, and alyssonoside inhibited the COX-catalyzed prostaglandin biosynthesis with the stronger inhibitory effects of COX-2 than COX-1. However, the inhibitory activity of the monomer compound is stronger than the extracts, as shown in Table 6. From the IC_50_ value, it can also be found that the value of the monomer compound is less than or equal to the value of the extract. At the same time, the value of the extracts is less than the granules of *C. nudiflora*, as shown in Table 7.

**TABLE 6:** Cyclooxygenase (COX-1 and COX-2) inhibition results.

Chemical compound	Concentration/(mg/ml)	Inhibition rate (%)	
		COX-1	COX-2
Verbascoside	0.1	102.81	108.27
2′-acetyl forsythiaside B	0.1	94.89	103.31
Forsythiaside B	0.1	91.04	104.38
Alyssonoside	0.1	80.54	103.15
Extract 1	0.1	79.84	99.38
Extract 2	0.1	81.00	100.93
Extract 3	0.1	76.31	104.34
Extract 4	0.1	−12.05	54.27
Extract 5	0.1	98.01	108.38
Extract 6	0.1	68.24	97.61

**TABLE 7:** IC_50_ effect on cyclooxygenase (COX-1 and COX-2).

Chemical compound	IC_50_/(mg/ml or μmol/L)	
	COX-1	COX-2
Granules of *C. nudiflora*	0.036	0.02
Extract 1	0.01	0.01
Extract 2	0.03	0.01
Extract 3	0.01	0.01
Extract 4	0.06	0.03
Extract 5	0.01	0.01
Extract 6	0.01	0.003
Verbascoside	0.02 or 31.14	0.002 or 3.14
2′-acetyl forsythiaside B	0.004 or 5.01	0.001 or 1.25
Forsythiaside B	0.01 or 13.22	0.002 or 2.64
Alyssonoside	0.03 or 38.94	0.008 or 10.40

## Discussion

The occurrence and development of many diseases are accompanied by the production of inflammation. Inflammation is not only related to diseases such as heart disease, atherosclerosis, and diabetes, but may also lead to tumors and brain diseases (Xu and Larbi, 2018). Many TCM has been reported to have good anti-inflammatory effects, for instance, *Astragalus membranaceus* (Fisch.) Bge*.*(Qi et al., 2017), *Paeonia lactiflora* Pallas. (Zhang and Wei, 2020), and so on. *C. nudiflora* has a nice anti-inflammatory effect (Lundgren et al., 1987) and is widely used in Hainan, China. However, the current research focuses on the extraction and separation of chemical components (Luo et al., 2015; Wang YL. et al., 2019), or the activity of monomer components (Huang et al., 2014; Wang HG. et al., 2019). These methods not only have the disadvantages of high cost and being time-consuming but also cannot reveal the multi-component effect of the *C. nudiflora*. TCM is a complex system with multiple components, multiple targets, and multiple pathways of action. The effect of TCM is usually due to the joint action of multiple ingredients. The spectrum–effect relationship is beneficial to find the main active ingredients of TCM. In this study, the spectrum–effect relationship between six extracts of *C. nudiflora* and the anti-inflammatory activity were explored through Pearson correlation analysis and the OPLS model.

The results of PCA and cluster analysis found that extracts 1, 2, 3, and 5 may contain many flavonoids, phenethyl alcohol glycosides, and phenolic acid compounds, and extracts 4 and 6 may contain a large amount of iridoids and other compounds. Then, it can be basically determined that the anti-inflammatory components of *C. nudiflora* are mainly phenethyl alcohol glycosides, flavonoids, and phenolic acid, followed by iridoid. Through the UPLC-Q/TOF-MS component identification, we speculate that catalpol (P1), caffeic acid (P2), protocatechuic acid (P9), 3,4-dihydroxybenzaldehyde (P10), forsythiaside E (P12), protocatechualdehyde isomers (P14), forsythiaside B (P15), rutin (P16), alyssonoside (P21), verbascoside (P22), 2′-acetyl forsythoside B (P24), and isorhamnetin (P32) are the material basis for the anti-inflammatory effects of *C. nudiflora*. Particularly, six compounds were identified as active substances with the greatest anti-inflammatory potential. These results indicate that the anti-inflammatory activity of *C. nudiflora* in inhibiting toe swelling of rats is not dominated by one compound but dominated by a combination of multiple components. According to reports, catalpol (Bhattamisra et al., 2019; Hu et al., 2019; Bi et al., 2020), caffeic acid (Zaitone et al., 2019; Paciello et al., 2020), protocatechuic acid (Tsai and Yin, 2012; Winter et al., 2017), 3,4-dihydroxybenzaldehyde (Chang et al., 2011; Zhang et al., 2020), rutin (Selloum et al., 2003; Wang et al., 2018; Budzynska et al., 2019), and isorhamnetin (Nam et al., 2015; Tsai et al., 2019) had anti-inflammatory effects.

In the process of inflammation in the body, cyclooxygenase (COX) is a key enzyme that can convert arachidonic acid metabolites into prostaglandin (PG) and other inflammatory and pain-causing substances, thereby causing inflammation (Kawahara et al., 2015). In this study, we have conducted studies on the inhibition of the activity of COX-1 and COX-2 on some of the selected compounds and six extracts. Another part of the selected compounds was searched for anti-inflammatory-related literature. The results show that the different extraction processes and active compounds of *C. nudiflora* have active effects on the biosynthesis of prostaglandin by cyclooxygenase (COX), and the inhibition of COX-2 is stronger than that of COX-1. COX-2 plays an important role in regulating the pathophysiological process of inflammation and is related to multiple inflammatory signal pathways (Malhotra et al., 2012; Mahesh et al., 2021). This provides a basis for us to further explore the anti-inflammatory mechanism of *C. nudiflora*. Through literature search and experiments, it was determined that each compound screened out had an anti-inflammatory effect. At the same time, we believe that the treatment of inflammation by *C. nudiflora* may be the result of multi-component interaction, and the synergy between the multi-components is the direction of our next study.

The anti-inflammatory mechanisms of natural products refer to the conduction of upstream signals of inflammation, such as NF-κB (Hamalainen et al., 2007; Korkina et al., 2011; Jiang et al., 2012), MAPK (Wang et al., 2015; Chi et al., 2016; Yang C. et al., 2020), Keap1/Nrf2/HO-1 (Wu et al., 2020), and JAK-STAT (Gong et al., 2020) inflammatory signaling pathways, thus regulating the secretion of various cheetification factors and inflammatory cytokines, for example, the production of ROS, NO, TNF-α, IL-1β, COX-2, IL-8, and IL-6. The anti-inflammatory action mechanism of *C. nudiflora* may be related to these inflammatory regulators, and the other specific mechanism of action remains to be further studied.

## Conclusion

In this study, the HPLC fingerprints of *C. nudiflora* were established, and 22 chromatographic peaks were selected. The spectrum–effect relationships between fingerprints and anti-inflammatory activities of *C. nudiflora* extracts were firstly studied. Twelve compounds from enethanol glycosides, flavonoids, iridoid, and phenolic acid compounds were determined to be the anti-inflammatory components of *C. nudiflora*. Particularly, six compounds were identified as active substances with the greatest anti-inflammatory potential. Moreover, some compounds were searched for anti-inflammatory literature, and the other parts were verified by experiments. This research helps to quickly screen the anti-inflammatory active ingredients of *C. nudiflora*, which can provide reference for the future study of active compounds of *C. nudiflora.*

## Data Availability

The original contributions presented in the study are included in the article/Supplementary Material. Further inquiries can be directed to the corresponding authors.

## References

[B1] Bhattamisra S. K. Yap K. H. Rao V. Choudhury H. (2019). Multiple Biological Effects of an Iridoid Glucoside, Catalpol and its Underlying Molecular Mechanisms. Biomolecules 10 (1), 32. 10.3390/biom10010032 PMC7023090 31878316

[B2] Bi F. Xu Y. Chen G. Wang P. (2020). Anti-inflammatory and Anti-endoplasmic Reticulum Stress Effects of Catalpol against Myocardial Ischemia-Reperfusion Injury in Streptozotocin-Induced Diabetic Rats. Acad. Bras Cienc 92 (4), e20191148. 10.1590/0001-3765202020191148 33237136

[B3] Budzynska B. Faggio C. Kruk-Slomka M. Samec D. Nabavi S. F. Sureda A. (2019). Rutin as Neuroprotective Agent: From Bench to Bedside. Curr. Med. Chem. 26 (27), 5152–5164. 10.2174/0929867324666171003114154 28971760

[B4] Cai J. P. Dong L. W. G. W. Liu M. S. (2012). Research Progress of Callicarpa Nudiflora. Mod. Drugs Clin. Med. 27 (1), 60–64.

[B5] Chang Z. Q. Gebru E. Lee S. P. Rhee M. H. Kim J. C. Cheng H. (2011). *In Vitro* antioxidant and Anti-inflammatory Activities of Protocatechualdehyde Isolated from Phellinus Gilvus. J. Nutr. Sci. Vitaminol (Tokyo) 57 (1), 118–122. 10.3177/jnsv.57.118 21512301

[B6] Chi G. Zhong W. Liu Y. Lu G. Lü H. Wang D. (2016). Isorhamnetin Protects Mice from Lipopolysaccharide-Induced Acute Lung Injury via the Inhibition of Inflammatory Responses. Inflamm. Res. 65 (1), 33–41. 10.1007/s00011-015-0887-9 26525359

[B7] Gong L. Yu L. Gong X. Wang C. Hu N. Dai X. (2020). Exploration of Anti-inflammatory Mechanism of Forsythiaside A and Forsythiaside B in CuSO4-Induced Inflammation in Zebrafish by Metabolomic and Proteomic Analyses. J. Neuroinflamm. 17 (1), 173. 10.1186/s12974-020-01855-9 PMC7271515 32493433

[B8] Hämäläinen M. Nieminen R. Vuorela P. Heinonen M. Moilanen E. (2007). Anti-inflammatory Effects of Flavonoids: Genistein, Kaempferol, Quercetin, and Daidzein Inhibit STAT-1 and NF-kappaB Activations, whereas Flavone, Isorhamnetin, Naringenin, and Pelargonidin Inhibit Only NF-kappaB Activation along with Their Inhibitory Effect on iNOS Expression and NO Production in Activated Macrophages. Mediators Inflamm. 2007, 45673. 10.1155/2007/45673 18274639 PMC2220047

[B9] Hu H. Wang C. Jin Y. Meng Q. Liu Q. Liu Z. (2019). Catalpol Inhibits Homocysteine-Induced Oxidation and Inflammation via Inhibiting Nox4/NF-κB and GRP78/PERK Pathways in Human Aorta Endothelial Cells. Inflammation 42 (1), 64–80. 10.1007/s10753-018-0873-9 30315526 PMC6394570

[B10] Huang B. Fu H. Z. Chen W. K. Luo Y. H. Ma S. C. (2014). Hepatoprotective Triterpenoid Saponins from Callicarpa Nudiflora. Chem. Pharm. Bull. (Tokyo) 62 (7), 695–699. 10.1248/cpb.c14-00159 24804828

[B11] Jiang W. L. Yong-Xu X. Zhang S. P. Zhu H. B. Jian-Hou H. (2012). Forsythoside B Protects against Experimental Sepsis by Modulating Inflammatory Factors. Phytother Res. 26 (7), 981–987. 10.1002/ptr.3668 22147417

[B12] Kawahara K. Hohjoh H. Inazumi T. Tsuchiya S. Sugimoto Y. (2015). Prostaglandin E2-Induced Inflammation: Relevance of Prostaglandin E Receptors. Biochim. Biophys. Acta 1851 (4), 414–421. 10.1016/j.bbalip.2014.07.008 25038274

[B13] Korkina L. Kostyuk V. De Luca C. Pastore S. (2011). Plant Phenylpropanoids as Emerging Anti-inflammatory Agents. Mini Rev. Med. Chem. 11 (10), 823–835. 10.2174/138955711796575489 21762105

[B14] Li Y. M. Yang Y. Yang Y. F. Li X. F. Kang X. D. Xiao J. P. (2020). Analysis of Different Extraction Process Components of Callicarpa Nudiflora Based on UPLC-QTOF MS/MS Technology. J. Chin. Med. Mater. 19 (11), 2707–2712. 10.13863/j.issn1001-4454.2020.11.019

[B15] Liang J. J. Xu K. Li L. F. (2009). Study of Total Flavonoids of Callicarpa Nudiflora on Ant-I Inflammatory and Hemostasis Effects. Mod. J. Integr. Tradit. Chin. West. Med. 18 (26), 3161–3162.

[B16] Lundgren G. Albrechtsen D. Brynger H. Flatmark A. Frödin L. Gäbel H. (1987). Role of HLA Matching and Pretransplant Blood Transfusions in Cyclosporine-Treated Recipients of Cadaveric Renal Allografts: 2- to 3-year Results. Transpl. Proc 19 (5), 3614–3618. 3313870

[B17] Luo Y. H. Ma S. C. Hu S. R. Fu H. Z. Zhou Z. Q. Chen W. K. (2015). Chemical Constituents from Callicarpa Nudiflora. Zhong Yao Cai 38 (11), 2306–2310. 10.13863/j.issn1001-4454.2015.11.017 27356380

[B18] Mahesh G. Anil Kumar K. Reddanna P. (2021). Overview on the Discovery and Development of Anti-inflammatory Drugs: Should the Focus Be on Synthesis or Degradation of PGE2? J. Inflamm. Res. 14, 253–263. 10.2147/JIR.S278514 33568930 PMC7868279

[B19] Malhotra S. Deshmukh S. S. Dastidar S. G. (2012). COX Inhibitors for Airway Inflammation. Expert Opin. Ther. Targets 16 (2), 195–207. 10.1517/14728222.2012.661416 22324934

[B20] Nam S. Y. Kim H. Y. Yoou M. S. Kim A. H. Park B. J. Jeong H. J. (2015). Anti-inflammatory Effects of Isoacteoside from Abeliophyllum Distichum. Immunopharmacol. Immunotoxicol. 37 (3), 258–264. 10.3109/08923973.2015.1026604 25975581

[B21] Paciello F. Di Pino A. Rolesi R. Troiani D. Paludetti G. Grassi C. (2020). Anti-Oxidant and Anti-inflammatory Effects of Caffeic Acid: *In Vivo* Evidences in a Model of Noise-Induced Hearing Loss. Food Chem. Toxicol. 143, 111555. 10.1016/j.fct.2020.111555 32640333

[B22] Qi Y. Gao F. Hou L. Wan C. (2017). Anti-Inflammatory and Immunostimulatory Activities of Astragalosides. Am. J. Chin. Med. 45 (6), 1157–1167. 10.1142/S0192415X1750063X 28830214

[B23] Selloum L. Bouriche H. Tigrine C. Boudoukha C. (2003). Anti-Inflammatory Effect of Rutin on Rat Paw Oedema, and on Neutrophils Chemotaxis and Degranulation. Exp. Toxicol. Pathol. 54 (4), 313–318. 10.1078/0940-2993-00260 12710715

[B24] Tsai S. J. Yin M. C. (2012). Anti-glycative and Anti-inflammatory Effects of Protocatechuic Acid in Brain of Mice Treated by D-Galactose. Food Chem. Toxicol. 50 (9), 3198–3205. 10.1016/j.fct.2012.05.056 22687555

[B25] Tsai S. W. Lin C. C. Lin S. C. Wang S. P. Yang D. H. (2019). Isorhamnetin Ameliorates Inflammatory Responses and Articular Cartilage Damage in the Rats of Monosodium Iodoacetate-Induced Osteoarthritis. Immunopharmacol. Immunotoxicol. 41 (4), 504–512. 10.1080/08923973.2019.1641723 31342791

[B26] Tu Y. Sun L. Guo M. Chen W. (2013). The Medicinal Uses of Callicarpa L. In Traditional Chinese Medicine: an Ethnopharmacological, Phytochemical and Pharmacological Review. J. Ethnopharmacol. 146 (2), 465–481. 10.1016/j.jep.2012.12.051 23313870

[B27] Wang Y. J. Yang Y. F. Gao D. (2008). Advances in the Research on the Chemical Constituents and Biological Activity of Callicarpa Nudiflora. Chin. Herbal Med. 39 (1), 133–138.

[B28] Wang H. Y. Wang H. Wang J. H. Wang Q. Ma Q. F. Chen Y. Y. (2015). Protocatechuic Acid Inhibits Inflammatory Responses in LPS-Stimulated BV2 Microglia via NF-κB and MAPKs Signaling Pathways. Neurochem. Res. 40 (8), 1655–1660. 10.1007/s11064-015-1646-6 26134310

[B29] Wang Z. Xia Q. Liu X. Liu W. Huang W. Mei X. (2018). Phytochemistry, Pharmacology, Quality Control and Future Research of Forsythia Suspensa (Thunb.) Vahl: A Review. J. Ethnopharmacol. 210, 318–339. 10.1016/j.jep.2017.08.040 28887216

[B30] Wang H. G. Luo F. K. Lei X. Yao Y. D. Liao G. C. Liu Z. Q. (2019a). 3, 4-Seco-Labdane Diterpenoids from the Leaves of Callicarpa Nudiflora with Anti-inflammatory Effects. Chin. J. Nat. Med. 17 (9), 707–712. 10.1016/S1875-5364(19)30085-8 31526506

[B31] Wang Y. L. Zhang Q. Yin S. J. Cai L. Yang Y. X. Liu W. J. (2019b). Screening of Blood-Activating Active Components from Danshen-Honghua Herbal Pair by Spectrum-Effect Relationship Analysis. Phytomedicine 54, 149–158. 10.1016/j.phymed.2018.09.176 30668364

[B32] Wang Y. (2014). Research Progress of the Clinical Application of Callicarpa Nudiflora. Mod. J. Integrated Chin. West. Med. 23 (35), 3983–3985.

[B33] Wei Q. Liu K. M. (2019). Research Progress on the Spectrum-Effect Relationship of Traditional Chinese Medicine. New Chin. Med. Clin. Pharmacol. 30 (5), 634–638. 10.19378/j.issn.1003-9783.2019.05.021

[B34] Winter A. N. Brenner M. C. Punessen N. Snodgrass M. Byars C. Arora Y. (2017). Comparison of the Neuroprotective and Anti-inflammatory Effects of the Anthocyanin Metabolites, Protocatechuic Acid and 4-Hydroxybenzoic Acid. Oxid. Med. Cel. Longev. 2017, 6297080. 10.1155/2017/6297080 PMC5504963 28740571

[B35] Wu A. Yang Z. Huang Y. Yuan H. Lin C. Wang T. (2020). Natural Phenylethanoid Glycosides Isolated from Callicarpa Kwangtungensis Suppressed Lipopolysaccharide-Mediated Inflammatory Response via Activating Keap1/Nrf2/HO-1 Pathway in RAW 264.7 Macrophages Cell. J. Ethnopharmacol. 258, 112857. 10.1016/j.jep.2020.112857 32298752

[B36] Xu W. Larbi A. (2018). Immunity and Inflammation: From Jekyll to Hyde. Exp. Gerontol. 107, 98–101. 10.1016/j.exger.2017.11.018 29187316

[B37] Yang Z. M. Gu Z. X. Yan X. J. (2015). Study on the Anti-inflammatory Activityof Callicarpa Nudiflora. Shi Zhen Guo Yi Guo Yao 26 (11), 2620–2622.

[B38] Yang C. Shi Z. You L. Du Y. Ni J. Yan D. (2020a). Neuroprotective Effect of Catalpol via Anti-oxidative, Anti-inflammatory, and Anti-apoptotic Mechanisms. Front. Pharmacol. 11, 690. 10.3389/fphar.2020.00690 32477145 PMC7240050

[B39] Yang J. Q. Li Y. M. Yang Y. F. Wu T. Li X. F. Kang X. D. (2020b). Chemical Constituents of Callicarpa Nudiflora Leaves. J. Chin. Med. Mater. 43 (7), 1617–1621. 10.13863/j.issn1001-4454.2020.07.015

[B40] Yang L. Jiang H. Wang S. Hou A. Man W. Zhang J. (2020c). Discovering the Major Antitussive, Expectorant, and Anti-inflammatory Bioactive Constituents in Tussilago Farfara L. Based on the Spectrum-Effect Relationship Combined with Chemometrics. Molecules 25 (3), 620. 10.3390/molecules25030620 PMC7037795 32023945

[B41] Yang Y. Li Z. Y. Shao J. J. Wang G. Wen R. Tian J. Z. (2021). Callicarpa Nudiflora Hook. & Arn.: A Comprehensive Review of its Phytochemistry and Pharmacology. J. Ethnopharmacol. 264, 113123. 10.1016/j.jep.2020.113123 32783986

[B42] Yi B. Zhang M. Hu Y. M. Chen T. Wang M. Y. Fegn S. X. (2019). Analysis of Constituents from Different Parts of Callicarpa Nudiflora by UPLC-Q-TOF-MS/MS. Zhongguo Zhong Yao Za Zhi 44 (21), 4661–4669. 10.19540/j.cnki.cjcmm.20190906.202 31872662

[B43] Zaitone S. A. Ahmed E. Elsherbiny N. M. Mehanna E. T. El-Kherbetawy M. K. ElSayed M. H. (2019). Caffeic Acid Improves Locomotor Activity and Lessens Inflammatory burden in a Mouse Model of Rotenone-Induced Nigral Neurodegeneration: Relevance to Parkinson's Disease Therapy. Pharmacol. Rep. 71 (1), 32–41. 10.1016/j.pharep.2018.08.004 30368226

[B44] Zeng L. J. Lin B. Song H. T. (2015). Progress in Study of Spectrum-Effect Relationship of Traditional Chinese Medicine and Discussions. Zhongguo Zhong Yao Za Zhi 40 (8), 1425–1432. 26281574

[B45] Zhang L. Wei W. (2020). Anti-inflammatory and Immunoregulatory Effects of Paeoniflorin and Total Glucosides of Paeony. Pharmacol. Ther. 207, 107452. 10.1016/j.pharmthera.2019.107452 31836457

[B46] Zhang L. Dong L. Huang J. Liu M. Li G. Zhang C. (2013). 3, 4-Seco-Labdane Diterpenoids from the Leaves of Callicarpa Nudiflora and Their Inhibitory Effects on Nitric Oxide Production. Fitoterapia 89, 218–223. 10.1016/j.fitote.2013.05.022 23742856

[B47] Zhang X. Y. Liu J. S. Gao S. M. (2019). Research Methods and Application Progress of Spectrum-Effect Relationship of Traditional Chinese Medicine. Chin. J. Tradit. Chin. Med. 44 (20), 4405–4411. 10.19540/j.cnki.cjcmm.20190429.201 31872625

[B48] Zhang Y. Lan M. Lü J. P. Li J. F. Zhang K. Y. Zhi H. (2020). Antioxidant, Anti-inflammatory and Cytotoxic Activities of Polyphenols Extracted from Chroogomphus rutilus. Chem. Biodivers 17 (1), e1900479. 10.1002/cbdv.201900479 31667925

[B49] Zhu C. S. Lin Z. J. Xiao M. L. Niu H. J. Zhang B. (2016). The Spectrum-Effect Relationship-A Rational Approach to Screening Effective Compounds, Reflecting the Internal Quality of Chinese Herbal Medicine. Chin. J. Nat. Med. 14 (3), 177–184. 10.1016/S1875-5364(16)30014-0 27025364

[B50] Zhu C. S. Nie A. Z. Wang X. (2019). Research Progress of Dose-Effect Relationship of Traditional Chinese Medicine. Chin. Herbal Med. 50 (7), 1708–1712.

