# Supplementary material for: Study on The Anti-Inflammatory Effects of Callicarpa nudiflora Based on The Spectrum–Effect Relationship
Source: Front Pharmacol. 2022 Jan 27;12:806808. doi: 10.3389/fphar.2021.806808 (PMC8829221; doi:10.3389/fphar.2021.806808)
Supplement: Supplementary file 1 [file Table1.DOCX]

**TABLE S1** Peak area, retention time and number of matches of 22 related peaks

| Peak number | Retention time | Extract 1 | Extract 2 | Extract 3 | Extract 4 | Extract 5 | Extract 6 | Matches |
| --- | --- | --- | --- | --- | --- | --- | --- | --- |
| P1 | 2.729 | 342.127 | 314.187 | 26.074 | 30.737 | 169.322 | 3.066 | 6 |
| P2 | 2.895 | 0.000 | 50.996 | 8.646 | 0.000 | 31.063 | 0.000 | 3 |
| P5 | 3.718 | 0.000 | 28.093 | 3.143 | 6.102 | 11.159 | 0.000 | 4 |
| P9 | 5.250 | 6.899 | 0.000 | 3.923 | 0.000 | 2.676 | 0.000 | 3 |
| P10 | 5.722 | 74.968 | 41.304 | 3.886 | 0.000 | 26.246 | 0.000 | 4 |
| P12 | 6.137 | 43.353 | 12.099 | 15.888 | 0.000 | 7.564 | 0.000 | 4 |
| P14 | 7.083 | 132.282 | 51.115 | 28.810 | 19.992 | 0.000 | 0.000 | 4 |
| P15 | 8.363 | 196.656 | 167.860 | 118.067 | 0.000 | 202.571 | 0.000 | 4 |
| P16 | 9.791 | 18.271 | 0.000 | 6.751 | 0.000 | 4.004 | 0.000 | 3 |
| P18 | 11.586 | 1085.883 | 1029.069 | 1136.899 | 6.616 | 662.935 | 1.340 | 6 |
| P19 | 12.796 | 74.698 | 54.589 | 55.704 | 0.000 | 32.285 | 0.000 | 4 |
| P21 | 13.909 | 59.450 | 0.000 | 19.097 | 10.542 | 0.000 | 0.000 | 3 |
| P22 | 14.952 | 318.200 | 162.745 | 158.693 | 0.000 | 104.078 | 0.000 | 4 |
| P24 | 17.689 | 20.079 | 0.000 | 14.579 | 0.000 | 3.989 | 0.000 | 3 |
| P26 | 18.495 | 45.767 | 52.260 | 58.142 | 0.000 | 30.596 | 1.869 | 5 |
| P27 | 19.630 | 20.476 | 16.766 | 10.538 | 0.000 | 9.576 | 0.000 | 4 |
| P28 | 20.189 | 12.654 | 9.907 | 11.056 | 2.686 | 6.469 | 0.859 | 6 |
| P29 | 20.919 | 19.095 | 24.241 | 18.867 | 0.000 | 10.900 | 1.186 | 5 |
| P31 | 22.779 | 19.175 | 19.777 | 20.672 | 0.000 | 10.995 | 2.057 | 5 |
| P32 | 23.333 | 3.990 | 0.000 | 0.000 | 0.000 | 2.005 | 0.248 | 3 |
| P33 | 24.149 | 9.663 | 10.209 | 10.477 | 0.000 | 4.537 | 1.570 | 5 |
| P34 | 25.018 | 32.473 | 38.848 | 31.316 | 0.000 | 20.288 | 2.404 | 5 |
